# Rostral Agranular Insular Cortex Lesion with Motor Cortex Stimulation Enhances Pain Modulation Effect on Neuropathic Pain Model

**DOI:** 10.1155/2016/3898924

**Published:** 2016-10-19

**Authors:** Hyun Ho Jung, Jaewoo Shin, Jinhyung Kim, Seung-Hee Ahn, Sung Eun Lee, Chin Su Koh, Jae Sung Cho, Chanho Kong, Hyung-Cheul Shin, Sung June Kim, Jin Woo Chang

**Affiliations:** ^1^Department of Neurosurgery, Yonsei University College of Medicine, Seoul, Republic of Korea; ^2^Brain Korea 21 PLUS Project for Medical Science and Brain Research Institute, Yonsei University College of Medicine, Seoul, Republic of Korea; ^3^Department of Electrical and Computer Engineering, College of Engineering, Seoul National University, Seoul, Republic of Korea; ^4^Department of Physiology, Hallym University College of Medicine, Chuncheon, Republic of Korea

## Abstract

It is well known that the insular cortex is involved in the processing of painful input. The aim of this study was to evaluate the pain modulation role of the insular cortex during motor cortex stimulation (MCS). After inducing neuropathic pain (NP) rat models by the spared nerve injury method, we made a lesion on the rostral agranular insular cortex (RAIC) unilaterally and compared behaviorally determined pain threshold and latency in 2 groups: Group A (NP + MCS; *n* = 7) and Group B (NP + RAIC lesion + MCS; *n* = 7). Also, we simultaneously recorded neuronal activity (NP; *n* = 9) in the thalamus of the ventral posterolateral nucleus and RAIC to evaluate electrophysiological changes from MCS. The pain threshold and tolerance latency increased in Group A with “MCS on” and in Group B with or without “MCS on.” Moreover, its increase in Group B with “MCS on” was more than that of Group B without MCS or of Group A, suggesting that MCS and RAIC lesioning are involved in pain modulation. Compared with the “MCS off” condition, the “MCS on” induced significant threshold changes in an electrophysiological study. Our data suggest that the RAIC has its own pain modulation effect, which is influenced by MCS.

## 1. Introduction

Neuropathic pain is a neurodegenerative disease, caused by lesion or dysfunction of the central or peripheral nervous system. It is one of the most difficult types of pain to control because it is a multidimensional clinical entity mediated by many different pathophysiological mechanisms [1–4]. Drug-refractory neuropathic pain has been treated with invasive treatments such as lesioning or electrical stimulation therapy in the central or peripheral nervous system. Because of advantages such as reversibility and adjustability, neuromodulation therapy has become more popular.

In 1991, Tsubokawa first reported the use of motor cortex stimulation (MCS) in a patient with chronic, drug-resistant neuropathic pain [5]. MCS was initially applied to central pain secondary to thalamic stroke, but, over time, its usage expanded to various other types of neuropathic pain. The clinical literature reveals that chronic MCS shows approximate 45 to 75% of pain control rate [6–10]. Thus, the MCS procedure was accepted as a promising therapy for patients with severe drug-refractory pain. However, despite the clinical use of MCS for pain modulation, the mechanisms underlying its effects remain unclear.

There were several imaging studies and electrophysiological investigations performed to solve the mechanism of MCS, and they showed that many brain structures are activated after MCS [11–13]. MCS was found to attenuate hyperactivity of thalamic neurons [5]. We have previously reported that MCS modulate pain-signaling pathways and suppress activation of the ventral posterolateral nucleus (VPL) [14]. The insular cortex, although not yet extensively explored, also showed clear involvement in pain perception through imaging studies using PET or fMRI. Within the insular cortex, in animal studies, the rostral anterior insular cortex (RAIC) has extensive reciprocal corticocortical connections which shows its involvement in multiple aspects of pain behavior [15]. Also after making a lesion in the RAIC, there were diminished pain-related behaviors in neuropathic models without lateralization, which shows clear evidence of the pain modulation role of RAIC [16].

The aim of this study was to evaluate the role of pain modulation in the RAIC during MCS.

## 2. Materials and Methods

### 2.1. Animals

All procedures were conducted according to the guidelines of the Ethical Committee of the International Association for the Study of Pain and approved by the Institution Animal Care and Use Committee (IACUC) of Yonsei University [17]. Male Sprague-Dawley rats (*n* = 23) weighing 180–200 g were used in this study. Three animals were housed per laboratory cage with food and water available ad libitum. Light was controlled under a 12 h light/dark (light on between 07:00 am and 19:00 pm) cycle. The temperature was maintained at 22 ± 2°C and relative humidity was at 55 ± 5%. Animals were allowed to acclimate for at least a week before surgery and behavioral testing. The behavior-based study of the MCS effect was observed in two animal groups: Group A, a neuropathic pain group (*n* = 7), and Group B, neuropathic pain + RAIC lesion group (*n* = 7). Furthermore, neuronal activity of MCS effect was measured electrophysiologically in the neuropathic pain group (*n* = 9).

### 2.2. Surgical Procedures

#### 2.2.1. Surgical Procedures for Pain Model

To induce neuropathic pain, we used the spared nerve injury (SNI) method [18]. Rats were deeply anesthetized with pentobarbital sodium (50 mg/kg, intraperitoneally), and the left sciatic nerve was exposed. Under a surgical microscope (Olympus, Tokyo, Japan), the three major divisions of the sciatic nerve were exposed, and the common peroneal and tibial nerves were completely ligated and transected. Hemostasis was completed, and the cut was closed with muscle and skin sutures.

#### 2.2.2. MCS Electrode Implant

For MCS, we used a custom-made liquid crystal polymer electrode [19]. One week after establishing the animal model for neuropathic pain, we measured the pain threshold to determine whether the neuropathic pain had been effectively induced. A detailed description of our behavior test for measuring pain threshold is in Section 2.3.1. After the behavior test, rats that did not exhibit a neuropathic pain response were excluded from this study. To implant the MCS electrode, rats were anesthetized with pentobarbital sodium (50 mg/Kg, intraperitoneally) and fixed with a stereotaxic frame (Narishige, Tokyo, Japan). The scalp was opened and the skull was exposed. To place the electrode on the left hindlimb area of the primary motor cortex [20], we made a rectangular hole (2.0 mm × 2.0 mm). The coordination was from −0.2 to +1.8 mm from the Bregma and from 0.5 to 2.5 mm from the midline. The electrode was placed in the epidural space, and the electrode was firmly fixed using bolts and glue. The scalp was secured with sutures after completing all procedures.

#### 2.2.3. RAIC Lesion

In Group B, prior to implanting the MCS electrode, we made a burr hole that allowed us to insert an electrode in the target site (RAIC, AP: anteroposterior direction: +1.0 mm from the Bregma, ML: midline: +4.5 mm right side, lateral from midline, and DV: dorsoventral direction: −6.0 mm from the dura mater) [16]. After inserting electrodes in the target coordinates, we delivered an electrical pulse of 0.1 mA for 10 seconds for the RAIC lesioning. Then, the lesioning electrode was removed and the MCS electrode was implanted.

### 2.3. Behavior Tests

**Figure 1:**
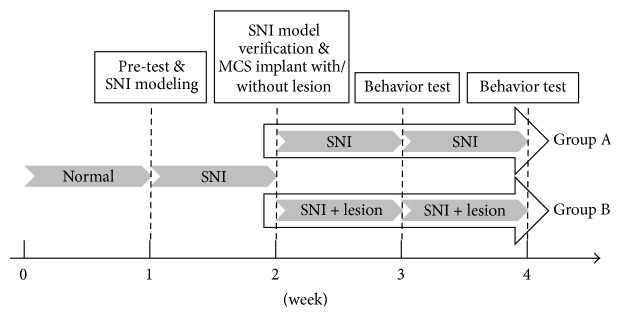
The timetable of spared nerve injury (SNI) modeling and behavioral test in two groups (Group A: neuropathic pain + motor cortex stimulation and Group B: neuropathic pain + rostral agranular insular cortex lesion + motor cortex stimulation).

The time table for SNI modeling and behavioral test in the two groups is presented in Figure 1.

#### 2.3.1. Measuring Tactile Threshold

Rats were placed inside acrylic cages (8 × 10 × 20 cm) on a wire mesh grid for measuring the mechanical threshold. After 30 minutes of adaptation, a series of von Frey filaments (0.4, 0.6, 1, 2, 4, 6, 8, and 15 g of bending force) were applied to the lateral edge of the left hind paw. We calculated the tactile threshold by using the up and down method [21].

#### 2.3.2. Measuring Response Latency

To measure the response latency, rats were placed in the same acrylic cages. After 30 minutes of adaptation, we applied painful stimulation to the left hindlimb, using a Plantar test unit (model 37370, Ugo Basile Biological Instruments, Comerio, VA, Italy) which measures the time by gradual application of strength automatically. When the rat initiated a withdrawal response, the Plantar test unit recorded the duration of resistance from stimulation and the value of final force. We measured the latency three times and used the average value for analysis.

#### 2.3.3. Behavioral Test Schedule and MCS Parameters

After 30 min of adaptation in the acryl cages, MCS was turned on (biphasic pulses of 65 Hz, 210 *μ*s, 80 *μ*A, for 30 min) using a stimulator (Model 2100, A-M Systems, Sequim, WA, USA). Behavioral tests were conducted at the following time points: before stimulation, 30 minutes after the start of stimulation, immediately after ceasing stimulation, and 5 times every 10 min.

### 2.4. Electrophysiology

We simultaneously recorded neuronal activity in the VPL of the thalamus and RAIC of NP model to compare the changes before and after MCS. Rats (*n* = 9), confirmed NP models after behavioral tests, were anesthetized with urethane (1.3 g/kg), and a microelectrode (573220, A-M Systems, Sequim, WA, USA) was inserted into the VPL and RAIC to obtain extracellular recordings of single unit activity. Two-channel array electrodes were positioned stereotactically in the VPL (ML: +2.8 mm; AP: −2.2 mm; DV: −6.0 mm from the Bregma) and the RAIC (AP: +1.0 mm; ML: +4.5 mm; DV: −6.0 mm from the Bregma). The neuronal activities were recorded for 5 minutes. During acquisition of the neural signal, mechanical stimulation, using 300 g of von Frey hair filaments, was applied to the rats' left hind paw area. Signals from the microelectrode were amplified (amplifier model 1700, A-M Systems, Sequim, WA, USA), and the signal was converted and transmitted to the recording system using an AD converter (Micro 1401, Cambridge Electronic Design Limited, Milton Road, Cambridge, UK). The data were stored by Spike 2 (Cambridge Electronic Design Limited, Milton Road, Cambridge, UK). Recorded waveforms were analyzed using Offline Sorter (Plexon Inc., USA), NeuroExplorer (NeuroExplorer Inc., USA).

Signal analysis was obtained for 20 sec before and after MCS. Because of firing differences in each region following MCS, the interval between the signal analyses was regulated.

### 2.5. Histological Verification of RAIC Lesion

**Figure 2:**
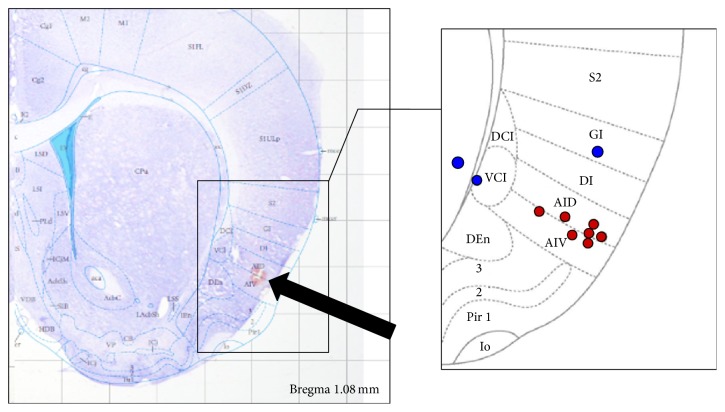
Histological verification of rostral agranular insular cortex (RAIC) lesions with fusing Mai atlas. Data from red dots (*n* = 7) were analyzed in this study. Blue dots were excluded from data analysis.

To verify the RAIC lesioning after completion of our experiments, rats were intracardially perfused with normal saline and fixed with 4% paraformaldehyde in PBS (pH = 7.4). The brain was carefully removed and prepared for frozen sectioning. Coronal sections of 30 *μ*m thickness were obtained using a microtome with deep freezer (Figure 2). The slices were dyed using cresyl violet. Microscopy images were obtained using a microscope (Olympus, Tokyo, Japan).

### 2.6. Statistical Analysis

Data are reported as mean ± SEM. Behavioral test data were analyzed using one-way and two-way analysis of variance (ANOVA) with Bonferroni's post hoc test. Electrophysiological data were evaluated using the Friedman test followed by Dunn's post hoc test. The *p* values of <.05 were considered significant. All statistical analyses were performed using SPSS (Version 20, SPSS Inc., Chicago, IL, USA).

## 3. Results

### 3.1. Changes of Mechanical Threshold in Groups A and B

**Figure 3:**
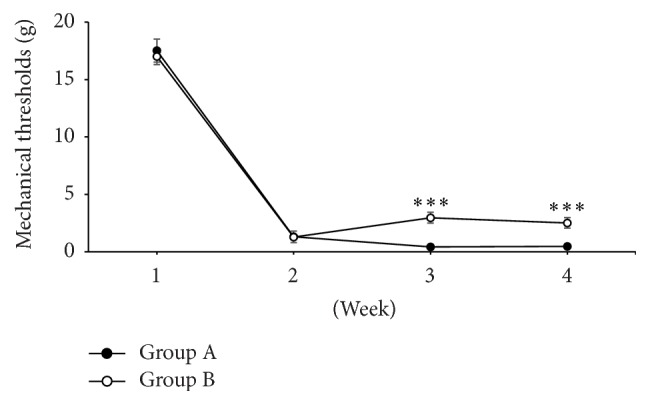
The change of mechanical thresholds was measured every week after pain modeling. Group B showed higher mechanical thresholds compared to Group A at 3rd and 4th weeks, which was statistically significant [two-way analysis of variance (ANOVA) with Bonferroni post hoc tests; ^*∗∗∗*^ *p* < .0001].

One week after pain modeling, we measured mechanical threshold in these rats. The average mechanical threshold was significantly decreased from 16.85 ± 0.50 to 1.2 ± 0.45 g (mean ± SEM) in Group B and from 17.00 ± 0.43 to 1.22 ± 0.38 g in Group A (Figure 3). After RAIC lesioning, in Group B, we measured the mechanical thresholds after the 2nd week. The average mechanical threshold of Group B was increased to 3.07 ± 0.53 g and this was also significantly higher (*p* < .001) than that of Group A (0.41 ± 0.09 g). At the 3rd week after modeling, the increased mechanical thresholds in Group B were maintained (2.75 ± 0.45 g), and the threshold was also significantly higher than that in Group A (0.46 ± 0.09 g, *p* < .001).

### 3.2. Changes in Mechanical Thresholds in Groups A and B with MCS

To examine the effect of MCS over time in each group, we measured pain thresholds in both groups with MCS at the 3rd week when the neuropathic pain model was established. Mechanical thresholds were measured in Groups A and B at regular time intervals. In both Groups A and B, the statistical significant changes of threshold were observed only during MCS on and immediate MCS off (Table 1). Compared to prestimulation value (Pre; 0.47 ± 0.90 g), mechanical threshold values were increased in Group A during MCS on (15 minutes; 3.85 ± 0.69 g) and immediate MCS off (30 minutes; 2.94 ± 0.42 g) and 10 minutes after MCS off (40 minutes; 2.27 ± 0.32 g). Similarly, compared to prestimulation value (Pre; 22.75 ± 0.45 g), mechanical threshold values were increased in Group B during MCS on (15 minutes; 10.08 ± 1.95 g) and immediate MCS off (30 minutes; 8.59 ± 2.45 g) and 10 minutes after MCS off (40 minutes; 8.42 ± 2.47 g).

**Figure 4:**
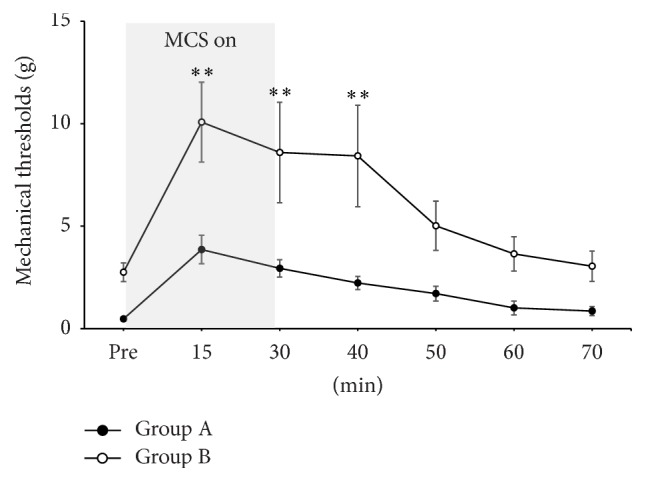
Changes of mechanical thresholds before, during, and after electrical stimulation of motor cortex stimulation (MCS) in Groups A and B. Two groups showed the increment of mechanical thresholds during MCS on, and these antinociceptive effects of MCS lasted for more than 30 minutes even though the electrical stimulation was off state. And the difference between mechanical thresholds of two groups was statistically significant until 40 minutes [two-way analysis of variance (ANOVA) with Bonferroni post hoc tests; ^*∗∗*^ *p* < .01].

In order to investigate the effect of RAIC lesion on mechanical thresholds over time, Groups A and B were compared at each time point. Statistically significant differences in threshold values between Groups A and B were observed at time points of 15, 30, and 40 minutes (Figure 4).

### 3.3. Latency

We measured pain response latency at 3 weeks after SNI modeling in both Groups A and B. In Group A, the baseline mechanical latency was 9.44 ± 0.37 sec, and the latency was significantly increased by MCS on, 15.37 ± 0.89 sec, which was statistically significant (*p* < .05). In Group B, the baseline latency was 15.96 ± 0.68 sec, and it increased to 19.00 ± 0.70 sec during MCS on, which was also statistically significant (*p* < .05).

**Figure 5:**
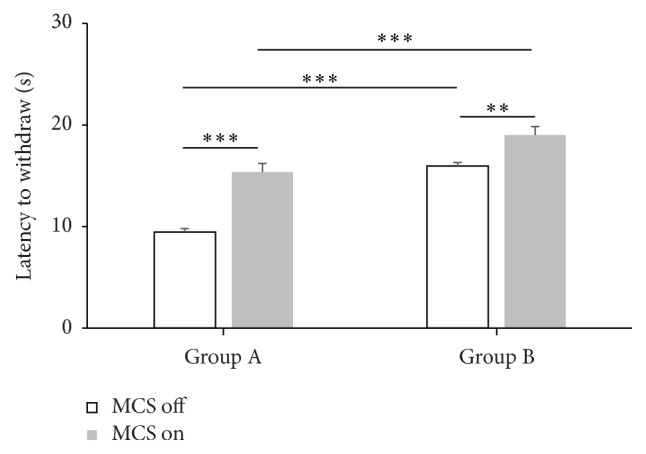
Overall changes of latency to withdrawal in Groups A and B with or without motor cortex stimulation (MCS). The latency increased with MCS on compared to MCS off in both Groups A and B which were statistically significant. During MCS off state, Group B showed longer latencies to withdrawal compared to Group A, which was statistically significant change. Also during MCS on state, latency increased in Group B with statistical significance [two-way analysis of variance (ANOVA) with Bonferroni post hoc tests; significant interaction between MCS and RAIC lesion factors *F* = 5.081, *p* = .0336]. ^*∗∗∗*^ *p* < .001 and ^*∗∗*^ *p* < .01.

In Group B without MCS, the latency showed significant increase compared with Group A without MCS. These findings were also noted after MCS on (Figure 5). Also, two-way analysis of variance showed significant interaction between MCS and RAIC lesion factors on withdrawal latency (Figure 5). Therefore, MCS with additional lesioning of the RAIC (Group B with MCS on) was more effective for pain suppression than MCS alone.

### 3.4. Neuronal Activity in the VPL and RAIC with MCS

**Figure 6:**
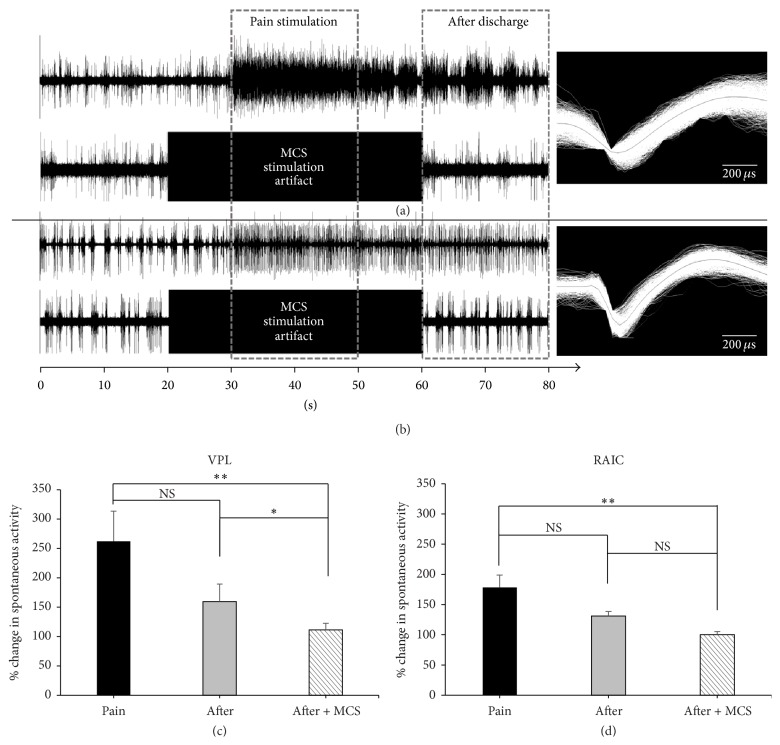
Electrophysiological recordings showing firing rate changes upon mechanical stimulation with 300 g von Frey filament and motor cortex stimulation. Firing rate at the ventral posterolateral nucleus (VPL) markedly increased upon pain stimulation and the trend persisted after discharge, but the postdischarge firing rate decreased when MCS was applied (a). Similarly, firing rate at the rostral agranular insular cortex (RAIC) markedly increased upon pain stimulation and the trend persisted after discharge, but the postdischarge firing rate decreased when MCS was applied (b). Each VPL and each RAIC wave form are presented on the right. Percentage changes in spontaneous neuronal activity, recorded from VPL (c) and RAIC (d), were decreased after MCS on state. And these changes were statistically significant compared with mechanical stimulation without MCS (*p* < .05). Statistical analysis was made using Friedman test followed by post hoc Dunn's multiple comparison test. ^*∗∗*^ *p* < .01; ^*∗*^ *p* < .05; NS = not  statistically significant.

Neuronal firing rate was rapidly increased in both VPL (245.9 ± 51.17%) and RAIC (171.7 ± 20.57%) upon mechanical pain stimulation and showed typical postdischarge patterns of neuropathic pain (Figures 6(a) and 6(b)). Without MCS, in both VPL and RAIC, percentage changes in spontaneous activity were not significantly different between pain stimulation (30–50 s; pain) and after discharge (60–80 s; after). Firing rates were significantly reduced in the postdischarge zone (60–80 s) with MCS on in both VPL (107.00 ± 11.43%) and RAIC (96.71 ± 6.00%) compared to firing rates during pain stimulation (30–50 s) with MCS off (VPL: 245.9 ± 51.17%; RAIC: 171.7 ± 20.57%; Figures 6(c) and 6(d)).

## 4. Discussion

The purpose of our study was to investigate the pain modulatory effect of MCS on the insular cortex, especially in the RAIC. The mechanism of MCS-induced pain modulation has still not been elucidated despite its clinical use. One of the most widely accepted hypotheses to explain the antinociceptive effect following MCS is that pain is modulated by descending inhibitory systems [2, 22–24]. The corticospinal tract from the motor cortex descends through the internal capsule and, after decussating in caudal medulla, reaches the spinal cord neurons in the anterior and posterior horn [25]. Because of a lack of direct projection from the primary motor cortex (M1) to superficial layers or the marginal zone of the dorsal horn, MCS may indirectly inhibit nociceptive inputs in the spinal cord [26]. Moreover, the motor cortex has diverse efferent projections to widely distributed cortical and subcortical areas. These structures include the thalamic nuclei, which receive strong projections from the motor cortex, an important site for sensory modulation [27]. The periaqueductal gray (PAG) system, coupled with the rostral ventromedial medulla (RVM), exhibits descending antinociceptive effects by activating the opioid system, and these two structures are connected with descending tracts [28]. Another MCS antinociception mechanism could be modulated by an ascending inhibitory system. The thalamus, activated by MCS, could inhibit nociceptive processing, but the specific nuclei affected by MCS and the source of altered inhibition are still debatable [29]. In an animal study of MCS, Cha et al. reported an enhanced inhibitory and antinociceptive input from the nucleus of zona incerta to the posterior thalamus [30].

Melzack and Casey suggested that the pain experience reflected interacting sensory, affective, and cognitive dimensions, which could influence each other [31]. In some imaging studies, attempts were made to determine the mechanism underlying the MCS. For example, using positron-emission tomography (PET), researchers have found that MCS was associated with increased blood flow in the orbitofrontal, subgenual anterior cingulate cortex, midcingulate cortex, insula cortices, thalamus, and brainstem [11–13]. Another PET study showed that the anterior midcingulate cortex and PAG were significantly correlated with the degree of clinical outcome of MCS, showing that these structures exhibited decreased exogenous ligand binding because of increased endogenous opioid secretion [32].

From previous imaging studies, one might infer that MCS could influence the insular cortex. However, there were no other experimental studies to implicate the role of the insular cortex in chronic stimulation of the motor cortex. The insular cortex is known as a multidimensional neuroanatomical convergence site for the integration of pain. By direct connection from the thalamoinsula pathway, pain information could be received at this site for sensory and affective integration. Historically, pain related to the insular cortex was only noted by asymbolia and pseudothalamic pain syndrome [33, 34]. Other evidence was from electrical stimulation of the posterior insula cortex, which produced pain with thermal stimuli in distinct sites on contralateral locations [35]. In animal studies, the RAIC showed somatic afferent pathways in relation to nociceptive input [15, 36, 37]. In addition, Coffeen et al. showed diminishment of neuropathic pain-related behaviors after lesioning in the RAIC of neuropathic model which was not in sham lesion, similar to our study. However, the MCS effect on the RAIC had not yet been demonstrated. Therefore, we used two groups of animals, each with an established neuropathic pain model, either without RAIC lesioning (Group A) or with lesioning (Group B), and compared their pain thresholds behaviorally and electrophysiologically. Upon mechanical stimulation, pain thresholds were significantly lower in Group A, which was what we expected from previous research. However, when we added MCS to both groups, Group B showed significant increase in threshold compared to Group A. These findings were also noted in pain latency from paw withdrawal tests. Therefore, we could assume that RAIC has its own pain modulation effect, and when adding MCS, the additional pain modulation effect could be shown. But, in our electrophysiological study, the percentages of changes in spontaneous activity were increased in both VPL and RAIC after mechanical stimulation with 300 g von Frey filament. Compared to the MCS off state, the changes of percentages after MCS on were noted in both regions (VPL and RAIC), which means the RAIC is also influenced by MCS. However, the quantitative influence of MCS on insular cortex is limited from our results. And though the RAIC has related to not only pain behavior but also anxiety behavior, we could make conclusion cautiously as above when we coupled it with our electrophysiological study.

In human studies, direct electrical stimulation of insular cortex during depth stereotactic EEG showed some degree of somatotopic organization [38]. But, in animal studies, the somatotopy related to pain process is not fully understood. Jasmin et al. showed a unique property of RAIC in that it could respond as both analgesic and hyperalgesic responses by selective modulation of GABA receptors [39]. The RAIC has multiple reciprocal connections with pain structures, such as orbital, infralimbic, and anterior cingulate cortices, rostroventral medulla, and periaqueductal gray matter [15]. In addition, caudal granular insular cortex (CGIC), about 4 mm caudal to RAIC, is also known as having a role in long term alleviation of allodynic pain modulation [40]. In this study, we did not compare the MCS effect on both the RAIC and the CGIC, so our findings have limitations on the information about MCS effect on the whole insular cortex. Additionally, the centromedian/parafascicular (CM/Pf) nuclei, which receive dense projection from the motor cortex, were inhibited by MCS, and these nuclei have interconnection with the limbic system. Therefore, our electrophysiological results could be the result of direct response to MCS or from indirect through CM/pf nuclei [41]. To clarify the source of the effect of MCS on RAIC, we will need to block the CM/Pf effect, which could also demonstrate the amount of MCS effect on RAIC.

## 5. Conclusions

The results in this work suggest that the RAIC is influenced by MCS and that lesioning RAIC could produce more pain reduction. Along with previous data, our findings may contribute to a better understanding of MCS effect and the role of RAIC in pain modulation.

## Figures and Tables

**Table 1:** Mechanical threshold measurement comparisons for Groups A and B at various time points: before (Pre), during (15 and 30 minutes), and after (40, 50, 60, and 70 minutes) motor cortex stimulation. Motor cortex stimulation began at 15 min time point.

Time (minute)	Mechanical thresholds (gram)	
	Group A	Group B
Pre	0.470 ± 0.090	2.750 ± 0.456
15	3.859 ± 0.698^*∗∗∗*^	10.080 ± 1.951^*∗∗*^
30	2.940 ± 0.423^*∗∗∗*^	8.596 ± 2.454^*∗*^
40	2.226 ± 0.321^*∗∗∗*^	8.428 ± 2.478^*∗*^
50	1.709 ± 0.360	5.015 ± 1.204
60	1.010 ± 0.339	3.646 ± 0.839
70	0.858 ± 0.218	3.045 ± 0.741

Comparisons among groups were made using repeated measures one-way analysis of variance (ANOVA). ^*∗∗∗*^ *p* < .001, ^*∗∗*^ *p* < .01, and ^*∗*^ *p* < .05 for comparisons.

## References

[1] Woolf C. J., Mannion R. J. (1999). Neuropathic pain: aetiology, symptoms, mechanisms, and management. *The Lancet*.

[2] Benedetti C. (1987). Intraspinal analgesia: an historical overview. *Acta anaesthesiologica Scandinavica. Supplementum*.

[3] Bouhassira D., Attal N., Fermanian J. (2004). Development and validation of the neuropathic pain symptom inventory. *Pain*.

[4] Finnerup N. B., Otto M., McQuay H. J., Jensen T. S., Sindrup S. H. (2005). Algorithm for neuropathic pain treatment: an evidence based proposal. *Pain*.

[5] Tsubokawa T., Katayama Y., Yamamoto T., Hirayama T., Koyama S. (1991). Chronic motor cortex stimulation for the treatment of central pain. *Acta Neurochirurgica Supplement (Wien)*.

[6] Rasche D., Ruppolt M., Stippich C., Unterberg A., Tronnier V. M. (2006). Motor cortex stimulation for long-term relief of chronic neuropathic pain: a 10 year experience. *Pain*.

[7] Cruccu G., Aziz T. Z., Garcia-Larrea L. (2007). EFNS guidelines on neurostimulation therapy for neuropathic pain. *European Journal of Neurology*.

[8] Lazorthes Y., Sol J. C., Fowo S., Roux F. E., Verdie J. C. (2007). Motor cortex stimulation for neuropathic pain. *Acta Neurochirurgica Supplement*.

[9] Saitoh Y., Yoshimine T. (2007). Stimulation of primary motor cortex for intractable deafferentation pain. *Acta Neurochirurgica, Supplementum*.

[10] Nguyen J.-P., Velasco F., Brugières P. (2008). Treatment of chronic neuropathic pain by motor cortex stimulation: results of a bicentric controlled crossover trial. *Brain Stimulation*.

[11] García-Larrea L., Peyron R., Mertens P. (1999). Electrical stimulation of motor cortex for pain control: a combined PET-scan and electrophysiological study. *Pain*.

[12] Peyron R., Laurent B., García-Larrea L. (2000). Functional imaging of brain responses to pain. A review and meta-analysis (2000). *Neurophysiologie Clinique*.

[13] Peyron R., Faillenot I., Mertens P., Laurent B., Garcia-Larrea L. (2007). Motor cortex stimulation in neuropathic pain. Correlations between analgesic effect and hemodynamic changes in the brain. A PET study. *NeuroImage*.

[14] Kim J., Ryu S. B., Lee S. E. (2016). Motor cortex stimulation and neuropathic pain: how does motor cortex stimulation affect pain-signaling pathways?. *Journal of Neurosurgery*.

[15] Jasmin L., Granato A., Ohara P. T. (2004). Rostral agranular insular cortex and pain areas of the central nervous system: a tract-tracing study in the rat. *Journal of Comparative Neurology*.

[16] Coffeen U., Manuel Ortega-Legaspi J., López-Muñoz F. J., Simón-Arceo K., Jaimes O., Pellicer F. (2011). Insular cortex lesion diminishes neuropathic and inflammatory pain-like behaviours. *European Journal of Pain*.

[17] Zimmermann M. (1983). Ethical guidelines for investigations of experimental pain in conscious animals. *Pain*.

[18] Decosterd I., Woolf C. J. (2000). Spared nerve injury: an animal model of persistent peripheral neuropathic pain. *Pain*.

[19] Lee S. E., Jun S. B., Lee H. J. (2012). A flexible depth probe using liquid crystal polymer. *IEEE Transactions on Biomedical Engineering*.

[20] Min K. S., Lee C. J., Jun S. B. (2014). A liquid crystal polymer-based neuromodulation system: an application on animal model of neuropathic pain. *Neuromodulation*.

[21] Chaplan S. R., Bach F. W., Pogrel J. W., Chung J. M., Yaksh T. L. (1994). Quantitative assessment of tactile allodynia in the rat paw. *Journal of Neuroscience Methods*.

[22] Yeung J. C., Rudy T. A. (1980). Multiplicative interaction between narcotic agonisms expressed at spinal and supraspinal sites of antinociceptive action as revealed by concurrent intrathecal and intracerebroventricular injections of morphine. *Journal of Pharmacology and Experimental Therapeutics*.

[23] Porreca F., Mosberg H. I., Omnaas J. R., Burks T. F., Cowan A. (1987). Supraspinal and spinal potency of selective opioid agonists in the mouse writhing test. *Journal of Pharmacology and Experimental Therapeutics*.

[24] Lipp J. (1991). Possible mechanisms of morphine analgesia. *Clinical Neuropharmacology*.

[25] Senapati A. K., Huntington P. J., Peng Y. B. (2005). Spinal dorsal horn neuron response to mechanical stimuli is decreased by electrical stimulation of the primary motor cortex. *Brain Research*.

[26] Stanley S. K., Ghanayem A. J., Voronov L. I. (2004). Flexion-extension response of the thoracolumbar spine under compressive follower preload. *Spine*.

[27] McAlonan K., Brown V. J. (2002). The thalamic reticular nucleus: more than a sensory nucleus?. *Neuroscientist*.

[28] Fonoff E. T., Dale C. S., Pagano R. L. (2009). Antinociception induced by epidural motor cortex stimulation in naive conscious rats is mediated by the opioid system. *Behavioural Brain Research*.

[29] Peyron R., Garcia-Larrea L., Deiber M. P. (1995). Electrical stimulation of precentral cortical area in the treatment of central pain: electrophysiological and PET study. *Pain*.

[30] Cha M., Ji Y., Masri R. (2013). Motor cortex stimulation activates the incertothalamic pathway in an animal model of spinal cord injury. *The Journal of Pain*.

[31] Melzack R., Casey K. L. (1968). *Sensory, Motivational, and Central Control Determinants of Pain: A New Conceptual Model*.

[32] Maarrawi J., Peyron R., Mertens P. (2007). Motor cortex stimulation for pain control induces changes in the endogenous opioid system. *Neurology*.

[33] Schmahmann J. D., Leifer D. (1992). Parietal pseudothalamic pain syndrome. Clinical features and anatomic correlates. *Archives of Neurology*.

[34] Augustine J. R. (1996). Circuitry and functional aspects of the insular lobe in primates including humans. *Brain Research Reviews*.

[35] Ostrowsky K., Magnin M., Ryvlin P., Isnard J., Guenot M., Mauguière F. (2002). Representation of pain and somatic sensation in the human insula: a study of responses to direct electrical cortical stimulation. *Cerebral Cortex*.

[36] Coffeen U., López-Avila A., Ortega-Legaspi J. M., del Ángel R., López-Muñoz F. J., Pellicer F. (2008). Dopamine receptors in the anterior insular cortex modulate long-term nociception in the rat. *European Journal of Pain*.

[37] Alvarez P., Dieb W., Hafidi A., Voisin D. L., Dallel R. (2009). Insular cortex representation of dynamic mechanical allodynia in trigeminal neuropathic rats. *Neurobiology of Disease*.

[38] Mazzola L., Isnard J., Peyron R., Guénot M., Mauguière F. (2009). Somatotopic organization of pain responses to direct electrical stimulation of the human insular cortex. *Pain*.

[39] Jasmin L., Rabkin S. D., Granato A., Boudah A., Ohara P. T. (2003). Analgesia and hyperalgesia from GABA-mediated modulation of the cerebral cortex. *Nature*.

[40] Benison A. M., Chumachenko S., Harrison J. A. (2011). Caudal granular insular cortex is sufficient and necessary for the long-term maintenance of allodynic behavior in the rat attributable to mononeuropathy. *The Journal of Neuroscience*.

[41] Pagano R. L., Fonoff E. T., Dale C. S., Ballester G., Teixeira M. J., Britto L. R. G. (2012). Motor cortex stimulation inhibits thalamic sensory neurons and enhances activity of PAG neurons: possible pathways for antinociception. *Pain*.

